# Modulation of Biological Membranes Using Small-Molecule Compounds to Counter Toxicity Caused by Amyloidogenic Proteins

**DOI:** 10.3390/membranes14110231

**Published:** 2024-11-06

**Authors:** Raina Marie Seychell, Adam El Saghir, Neville Vassallo

**Affiliations:** 1 Department of Physiology and Biochemistry, Faculty of Medicine and Surgery, University of Malta, MSD 2080 Msida, Malta; 2 Centre for Molecular Medicine and Biobanking, University of Malta, MSD 2080 Msida, Malta

**Keywords:** protein-misfolding diseases, amyloid, protein aggregation, lipid membranes, small molecules, therapeutics

## Abstract

The transition of peptides or proteins along a misfolding continuum from soluble functional states to pathological aggregates, to ultimately deposit as amyloid fibrils, is a process that underlies an expanding group of human diseases—collectively known as protein-misfolding disorders (PMDs). These include common and debilitating conditions, such as Alzheimer’s disease, Parkinson’s disease, and type-2 diabetes. Compelling evidence has emerged that the complex interplay between the misfolded proteins and biological membranes is a key determinant of the pathogenic mechanisms by which harmful amyloid entities are formed and exert their cytotoxicity. Most efforts thus far to develop disease-modifying treatments for PMDs have largely focused on anti-aggregation strategies: to neutralise, or prevent the formation of, toxic amyloid species. Herein, we review the critical role of the phospholipid membrane in mediating and enabling amyloid pathogenicity. We consequently propose that the development of small molecules, which have the potential to uniquely modify the physicochemical properties of the membrane and make it more resilient against damage by misfolded proteins, could provide a novel therapeutic approach in PMDs. By way of an example, natural compounds shown to intercalate into lipid bilayers and inhibit amyloid–lipid interactions, such as the aminosterols, squalamine and trodusquamine, cholesterol, ubiquinone, and select polyphenols, are discussed. Such a strategy would provide a novel approach to counter a wide range of toxic biomolecules implicit in numerous human amyloid pathologies.

## 1. Introduction

Protein-misfolding disorders (PMDs) are a growing challenge in modern medicine, marked by their complex pathogenesis, lack of effective treatments and increasing prevalence. These clinical syndromes, broadly described as proteinopathies, are characterised by aberrant protein folding, self-assembly and, ultimately, deposition of large insoluble amyloid assemblies. In healthy living systems, proteins adopt various conformational states, which are governed by complex kinetic and thermodynamic equilibria, together with intricate quality control mechanisms, regulating and maintaining proteome integrity and cellular health. However, in the event of erroneous folding, some proteins can acquire toxic, non-native conformations, leading to cellular damage and disease progression [1,2].

A large group of human disorders have been linked to this toxic sequence of protein misfolding and amyloid deposition. They include degenerative, and highly debilitating diseases that burden ageing populations, most notably Alzheimer’s disease (AD), Parkinson’s disease (PD), type-2 diabetes mellitus (T2DM), as well as a number of systemic conditions [1,3]. A summarised list of amyloidogenic proteins associated with human diseases is provided in Table 1. Despite the absence of similarities amongst the amyloid-associated proteins in the primary sequence or function, they all share a common pathogenic pathway involving the restructuring of proteins into pathological aggregates, with eventual formation of amyloid fibrils [4,5].

Amyloidogenesis commonly involves what are referred to as intrinsically disordered peptides (IDPs). The amyloid-β (Aβ), amylin (also known as the human islet amyloid polypeptide, hIAPP), α-synuclein (α-syn), tau and prion proteins all belong to the amyloidogenic IDP family; these species are largely unstructured in their native form and can adopt a continuum of transient conformations in the aqueous phase [7]. The course of fibril assembly by amyloid proteins follows a nucleation-dependent polymerisation pathway. The kinetics of amyloid formation generally exhibit a sigmoidal pattern, comprising three distinct phases of growth; the initial lag phase is followed by rapid growth and towards completion, the curve reaches a plateau [8]. The initial step in this process entails the formation of transient oligomeric species, which are dynamic and heterogeneous in structure [9]. The lag phase is predominantly populated by monomeric precursors, which aggregate into critical nuclei via distinct phases of growth; the initial lag phase is followed by rapid growth and towards completion, the curve reaches a plateau. The growth-competent nuclei act as a template for the accumulation of additional protein molecules [10,11]. During the elongation phase, monomers and small oligomeric assemblies continue to interact, while the prefibrillar aggregates rapidly extend and develop into ordered protofibrils. Finally, the saturation phase is reached, wherein the slope of the kinetic curve reaches a plateau due to monomer consumption and protofibrils assemble into end-stage fibrils [5,11]. The resulting amyloid fibrils exhibit a common structural motif in the fibrillar state, described as the cross-β-spine, comprised of a highly ordered structure rich in intermolecular β-strands arranged perpendicular to the main fibril axis [1,12].

In this complicated cascade of events, soluble oligomeric assemblies are generated, which are generally described as the primary cytotoxic species along the aggregation pathway. The mechanisms of oligomer-mediated cellular toxicity include the generation of metal and ionic dyshomeostasis, induction of elevated calcium levels, perturbation of mitochondrial function and generation of reactive oxygen species (ROS), amongst others [13]. Compared to amyloid fibrils, these low-molecular-weight conformers possess distinct molecular features that enhance their potential to induce toxicity, such as their small size, exposed hydrophobic patches and unsaturated edge-strands [14,15]. These physicochemical features allow promiscuity in interactions with a range of cellular components. Particularly, oligomers possess a high affinity for biological membranes and have the capacity to insert into lipid bilayers due to their amphipathic nature [7,13]. Furthermore, accumulating evidence indicates that amyloid formation and cytotoxicity are membrane-assisted processes, with lipid bilayers presenting a platform for initiating the conversion of the IDPs into β-sheet-rich aggregates that are conductive to fibril formation [16,17].

## 2. Toxic Interplay Between Amyloid Proteins and Membranes

Lipid membranes serve as a highly complex hydrophobic boundary between the homeostatic intracellular space and the highly fluctuating extracellular environment, as well as providing a functional barrier between subcellular compartments. Phospholipids are the primary constituent of biological membranes, together with sphingolipids and sterols [18,19]. Due to the inherent complexity of membranes, many researchers utilise structurally and compositionally simplified membrane models, including monolayers, micelles, liposomes, and supported lipid bilayers (SBLMs), to probe the interactions between amyloidogenic assemblies and membranes [20].

The interaction of amyloid proteins with phospholipid bilayers is essentially a two-way interaction [21,22]. On the one hand, the detrimental effects of amyloid aggregates are strongly associated with their interactions with biomembranes [23,24,25,26]. On the other hand, lipid bilayers can also fulfil a catalytic role in the generation of fibrillar amyloids, with a growing body of research suggesting that the toxic cascade of amyloid assembly commences at membrane surfaces [27,28,29,30,31]. Hence, the protein–lipid environment plays a fundamental role in the kinetics of both fibrillogenesis and cytotoxicity, with the interaction between amyloid-forming peptides and lipid membranes leading to a mutually destructive perturbation of both parties.

The bilayer composition exerts a profound influence on the routes of adsorption, incorporation, orientation, structure, and assembly pathways of amyloid peptides [32,33]. Most notably, negatively charged headgroups of phospholipids, like phosphatidic acid (PA), phosphatidylserine (PS), and phosphatidylinositol (PI), strongly favour aggregation on membrane surfaces. The influence of anionic phospholipids on membrane binding, aggregation and disruption has been well documented for the hIAPP [31,34,35], htt [36], α-syn [37,38], Aβ [39,40], and tau [41,42] proteins. Other membrane components are also important for amyloid–membrane interactions [40]. For example, variations in the relative size of the phospholipid headgroup and acyl chains can give rise to local membrane defects and intrinsic curvature, which in turn influence the extent of the peptide alignment and adsorption at the membrane surface [43]. Smaller headgroups accelerate the kinetics of Aβ40 fibril assembly, shortening the lag time for primary nucleation [40]. Recent evidence indicates that the lipid bilayer thickness may also influence Aβ membrane-associated aggregation. Interestingly, membrane bilayers with the shorter-chain lipid dilauroyl phosphatidylcholine (DLPC) remodelled preformed Aβ fibrils into toxic misfolded aggregates, suggesting that pathologically thin bilayers may have a role in Aβ aggregation [44]. Experiments using quartz crystal microbalance–dissipation (QCM-D) confirmed that the hydrophobic thickness of supported bilayers impacts the membrane remodelling triggered upon adsorption of Aβ [45].

The presence of a high curvature [46,47,48] and packing defects [49,50,51] are reported to facilitate membrane association and permeabilisation events. Aβ has been reported to preferentially associate with small unilamellar vesicles (SUVs, ~15–50 nm) characterised by higher surface curvature, and such architecture accelerates the nucleation step [47,52]. Due to the presence of more water-accessible hydrophobic regions, SUVs most likely trigger the aggregation process by concentrating Aβ monomers sufficiently to initiate nucleation [47,48]. The binding of Aβ to membranes is also profoundly influenced by lipid packing defects and electrostatic interactions with the lipid headgroups. Packing defects are induced by the incorporation of lipids, which do not possess a cylindrical morphology. Specifically, no significant binding was observed between Aβ40 and membranes composed of 1,2-dioleoyl-sn-glycero-3-phospho-(1′-rac-glycerol) (DOPC; a cylindrical and zwitterionic lipid), in contrast to significant early binding events with 1,2-dioleoyl-sn-glycero-3-phospho-(1′-rac-glycerol) (DOPG; a cylindrical and anionic lipid) and PI (an inverted conical and anionic lipid) [51]. αS has also been described to possess an affinity for lipid-packing defects present on highly curved lipid surfaces as well as in planar membranes with cone-shaped lipids [50,53,54]. hIAPP is described as a curvature-sensing and curvature-inducing peptide possessing an affinity for high curvature regions of bilayers and, in turn, causing further negative curvature strain within membranes. Supporting this, hIAPP is demonstrated to preferentially localise to mitochondrial cristae, which are highly curved structures, rich in PE and CL [46].

Membrane destabilisation is described as a key patho-mechanism for PMDs. Compelling evidence provides support for the hypothesis that cytotoxicity is predominantly triggered by the interaction of prefibrillar amyloids with cellular lipid bilayers. This interaction leads to bilayer perturbation, inducing membrane leakage, disrupting ionic homeostasis, and triggering cellular dysfunction, oxidative stress and apoptosis [55,56,57,58,59]. Although it is generally accepted that the disruptive effects of amyloid-forming proteins are strongly correlated with their ability to interact with and disturb biological membranes, the exact molecular mechanisms governing these deleterious interactions remain elusive [60]. The three primary mechanistic models that have been proposed to describe the mechanisms by which amyloid assemblies cause membrane damage are (i) the creation of stable transmembrane protein pores, (ii) the destabilisation of the bilayer through a “carpet model”, and (iii) the removal of lipids via detergent-like membrane dissolution [60,61]. Furthermore, the insertion of amyloid peptides into lipid bilayers can disrupt the structural integrity of the membrane by inducing local bending deformations and curvature stress [32,62,63]. In addition to the interactions of early aggregate species with membranes, one should also consider the effects of amyloid fibrils on bilayer integrity. A fibrillar amyloid induces membrane damage via its elongation on the membrane surface through secondary nucleation, which has been especially well documented for hIAPP [64,65]. It is highly probable that the mechanisms in operation are not mutually exclusive; instead, membrane disruption is instigated by the combined effects of multiple models [61,66].

## 3. Small-Molecule Amyloid Inhibitors: The Case for Modulating Lipid Membranes

The complex pathophysiology of amyloid diseases, involving a highly heterogenous population of protein aggregate species that can exert their cytotoxic effects through a wide variety mechanisms, renders the development of effective treatments for these disorders exceptionally challenging. As a result, different therapeutic modalities have been explored to address these disorders, with most efforts to date being focused on the discovery of potent anti-aggregation agents. In this regard, several natural and synthetic small molecules have been identified that can target different aspects of the aggregation cascade, including stabilisation of the native conformation; inhibition of monomer aggregation into toxic oligomers; redirecting the aggregation pathway into a non-toxic oligomer population; structural modification of the oligomeric conformers; and accelerating aggregation into high-molecular-weight fibrillar species, thereby reducing the toxic oligomer population [67,68,69,70,71]. Yet other approaches focus on enhancing the clearance of misassembled and misfolded proteins [13,72]. Despite promising findings in laboratory settings, however, clinical outcomes involving anti-amyloid strategies have been largely disappointing. In this respect, the Food and Drug Administration (FDA) approvals of the antibodies lecanemab and aducanumab for the treatment of AD have arguably bolstered support for anti-amyloid-focused therapeutic strategies [73].

**Figure 1:**
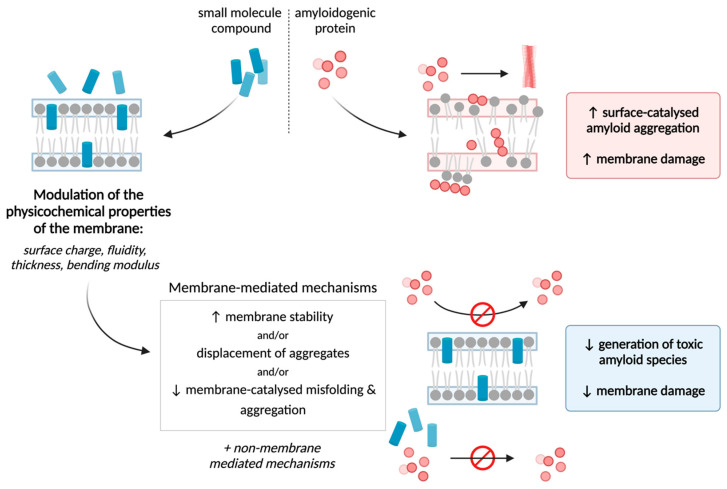
Key protective mechanisms of membrane-mediated protection against amyloid toxicity by small molecules. The schematic illustrates multiple mechanisms through which therapeutic compounds can be exploited to modulate phospholipid membranes and confer protection against bilayer disruption by toxic amyloid assemblies. Specifically, by altering the physicochemical properties of phospholipid bilayers, these small molecules can impede the misfolding, accumulation and insertion events of amyloidogenic peptides, thereby mitigating amyloid toxicity.

**Figure 2:**
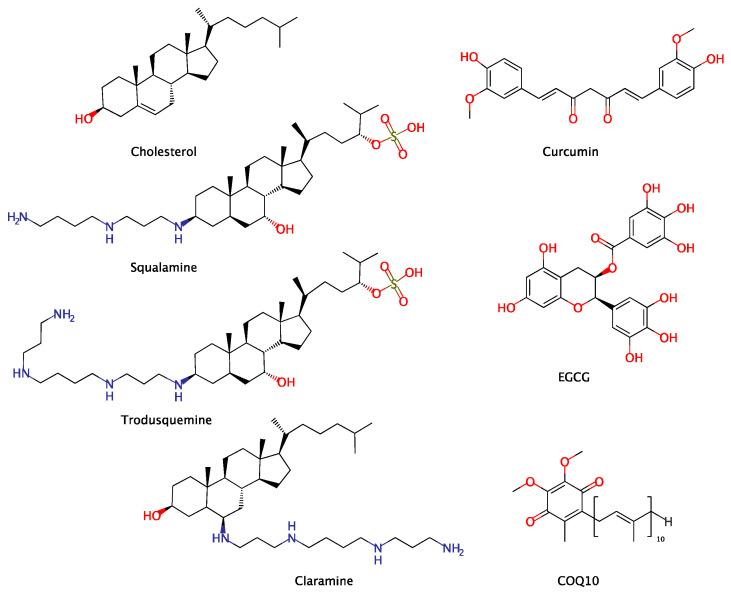
Structures of the natural compounds reviewed for their membrane-mediated protective activities against amyloid toxicity.

As discussed above, the toxicity of aggregates derived from amyloidogenic proteins is largely mediated by their interactions with the cellular *milieu*. In particular, direct association with, and perturbation of, biomembranes are the mechanisms that appear to be most commonly identified when amyloid toxicity is addressed in vitro. Further, amyloid protein–lipid interactions may instigate misfolding and assembly of the intrinsically disordered peptides, thereby potentially exacerbating aggregation. It follows from the above considerations that modulating the physical state of lipid membranes might act as an effective countermeasure against the membrane-active properties of the misfolded proteins. Many small-molecule compounds, both natural and synthetic, are known to interact with and insert into biological membranes, thereby inducing alterations to the physicochemical properties of the lipidic bilayer; for example, changes in the membrane surface charge, membrane fluidity, bilayer thickness, and membrane bending stiffness [74,75]. Thus, in addition to “classical” inhibitory mechanisms of protein aggregation, anti-amyloid agents might also include those compounds that uniquely intercalate into lipid bilayers to safeguard membranes from toxic biomolecules implicit in numerous human proteinopathies (Figure 1). The following section shall explore a number of naturally derived molecules that have been demonstrated to exhibit this mechanism. Such molecules include aminosterols, cholesterol and polyphenolic compounds (Figure 2).

### 3.1. Aminosterols

#### 3.1.1. Squalamine and Trodusquemine

Squalamine (SQ) and Trodusquemine (TRO) represent an intriguing pair of natural aminosterols, originally isolated from the dogfish shark, *Squalus acanthias.* As will be discussed in more detail below, they have recently been extensively investigated for their potential in combating amyloid disorders through their ability to bind biological membranes [76]. Both SQ and TRO have successfully completed phase-1 clinical trials, demonstrating favourable safety and tolerability profiles (NCT00606112 and NCT00139282, respectively).

SQ and TRO are structurally very similar, and they are both cationic amphipathic aminosterols. Thus, they share a hydrophobic sterol domain with a sulphated side chain whilst exhibiting distinct hydrophilic polyamine moieties: spermine (possessing four amine groups) in TRO and spermidine (possessing three amine groups) in SQ. This results in TRO carrying a greater net positive charge (+3) compared to SQ (+2) at a physiological pH [77,78]. The cationic polyamine side chains confer the ability to neutralise the negative charges on the inner leaflet of eukaryotic membranes, consequently displacing proteins electrostatically bound to the bilayer [79]. In agreement with this mechanism, the difference of a single positive charge between the two aminosterols appears to exert a significant impact on their respective efficacies, with TRO demonstrating a stronger protective effect relative to equivalent concentrations of SQ. Moreover, the architecture of these cationic steroids, featuring a negatively charged sulphate group on one side of the sterol core and a positively charged polyamine on the other, is a configuration known to significantly stabilise lipid membranes [78].

Nuclear magnetic resonance (NMR) experiments suggest that SQ effectively out-competes α-syn for binding sites on lipid vesicles, thereby mitigating α-syn aggregation and reducing oligomer-mediated toxicity [80,81]. A decline in protein–vesicle interactions was observed without any direct binding between SQ and α-syn—thus favouring the notion that the protective effects of the compound are primarily mediated via its membrane interactions. Competitive displacement of α-syn was further evidenced by a dose-dependent reduction in the α-helical content of α-syn upon incubation with lipid vesicles and SQ. In a *Caenorhabditis elegans* (*C. elegans*) model of PD, SQ significantly reduced the formation of intracellular inclusions and improved motility without affecting α-syn expression [80]. Moreover, in PD mouse models and aged wild-type mice, SQ has been shown to restore enteric neuron excitability and restore colonic motility [82,83]. Beyond these cellular and animal models, SQ has demonstrated notable efficacy in human clinical trials, including a phase-2b clinical trial (KARMET, NCT03781791), showing improvements in PD-related constipation, hallucinations, and dementia [84].

The other aminosterol, TRO, is notable for its efficacy at lower doses and its ability to cross the blood–brain barrier, enhancing its therapeutic potential in neurodegenerative diseases [76,85,86]. Trodusquemine stably integrates with high affinity into the interface between the hydrophilic and hydrophobic portions of the bilayer, exposing both charged sulphate and spermine groups to the aqueous environment. This insertion induces three key physicochemical alterations to the bilayer: a reduction in the total negative charge, an increased mechanical resistance to indentation, and a reorganisation of the lipid constituents [77]. Overall, these modifications “stiffen” biological membranes, making them more resistant to perturbation by misfolded protein oligomers [78]. Perni and co-workers [85] described a dose-dependent inhibition of lipid-induced α-syn assembly by TRO in the presence of anionic 1,2-dimyristoyl-sn-glycero-3-phospho-L-serine (DMPS) vesicles. While in the absence of TRO all the α-syn was bound to the vesicles in an α-helical conformation, circular dichroism (CD) experiments revealed that TRO shifted the signal of α-syn to that characteristic of a random coil, indicating the displacement of the protein, akin to SQ. Data suggest that the inhibitory mechanism exhibited by TRO appears to involve not only membrane displacement of monomeric α-syn, as seen with SQ, but also interaction with intermediate aggregation species. While TRO was reported to not influence fibril elongation events, this aminosterol significantly decreased the rate of secondary nucleation of α-syn, implying a selective mechanism targeting the early stages of amyloidogenesis [85,87]. Furthermore, confocal microscopy revealed that TRO inhibited α-syn oligomer toxicity in human neuroblastoma (SH-SY5Y) cells; this is attributed to the ability of TRO to displace and directly interact with oligomers, as suggested by the synthetic vesicle investigations. In the same investigation [85], Perni et al. also conducted in vivo studies using *C. elegans* models of PD and reported reduced intracellular inclusions and improved motility and longevity upon TRO administration.

The membrane-safeguarding effects of TRO extend beyond α-syn, as the aminosterol has also been shown to suppress the toxicity of oligomers of Aβ peptides (Aβ_42_ and Aβ_40_) and of the amyloidogenic N-terminal domain of the *Escherichia coli* (*E. coli*) HypF protein (HypF-N). At protective concentrations, TRO completely prevented oligomers from binding to the bilayer [87,88]. Interestingly, while TRO slowed down the lipid-induced aggregation of α-syn, it accelerated the amyloid fibril formation of Aβ_42_ by predominantly enhancing secondary nucleation events. This acceleration shifted the assembly process towards the generation of mature fibrils, hence depleting cytotoxic oligomeric intermediates. These observations were also replicated in vivo, wherein TRO administration in *C. elegans* models of AD was associated with increased aggregate formation but reduced Aβ_42_-induced toxicity [89].

In conclusion, although α-syn, amyloid-β, and HypF-N follow distinct aggregation kinetics, both aminosterols demonstrate efficacy in suppressing amyloid toxicity through a shared mechanism of outcompeting and displacing oligomeric aggregates from cell membranes.

#### 3.1.2. Claramine

Claramine, a blood–brain barrier-permeable steroid polyamine, is structurally analogous to TRO, having a spermine side chain attached to a sterol ring. Once again, this configuration enables the interaction with cellular membranes and modulation of their physicochemical properties. In a recent investigation by Kreiser et al. [90], claramine was found to neutralise the cytotoxicity of two pore-forming biological toxins, namely melittin from honeybee venom and α-hemolysin from *Staphylococcus aureus*. Claramine effectively prevented these porating agents from binding to cell membranes, without any detectable alterations to their overall structures. Importantly, this illustrates that the protective effects of claramine are mediated primarily through its modulation of membrane properties, rather than by direct interaction with the pore-forming proteins themselves, akin to the other aminosterols discussed [90]. With regards to amyloid proteins, it was reported that claramine effectively inhibited the aggregation of α-syn. In a well-established *C. elegans* model of PD, claramine administration resulted in a marked reduction in α-syn inclusions, accompanied by improved motility. These effects were attributed to the ability of this sterol to alter the phase behaviour of α-syn, particularly by slowing the rate of primary nucleation, a critical initial step in the aggregation process [91].

Based on these collective observations, it can be inferred that the effects of aminosterols in promoting membrane resilience against permeabilising biological toxins and misfolded oligomers can be ascribed mainly to their ability to regulate the physicochemical properties of cell membranes [76]. It is therefore tempting to speculate regarding a broad protective mechanism, which might be applicable to various human pathologies where membrane integrity is compromised. Additionally, the proven safety profiles and favourable pharmacokinetic properties of aminosterols, as supported by various clinical trials, further support their potential as therapeutic agents.

### 3.2. Cholesterol

Cholesterol is a physiologically very important component of lipid membranes that plays a key role in modulating membrane fluidity, permeability, and hydrophobicity [92,93]. This amphipathic molecule is composed of three well-defined regions: a small polar hydroxyl group, a rigid steroid ring, and a flexible iso-octyl side chain. Within membrane bilayers, cholesterol intercalates such that the polar hydroxyl group is positioned between the phospholipid polar head groups while the plate-like steroid ring extends into the hydrophobic core of the bilayer, wherein it imparts a condensing effect on the membrane [92,94]. Cholesterol fulfils a particularly important role in the brain, which accounts for 20–25% of the total body cholesterol [95,96]. Dysregulation of cholesterol homeostasis, particularly in the neuronal plasma membrane, has been implicated in synaptic loss, impaired synaptic plasticity, and the pathological changes observed in various neurodegenerative diseases, including AD, PD, and Huntington’s disease (HD) [96,97,98].

The interplay between cholesterol, protein aggregation and aggregate toxicity is complex and conflicting results have been reported [61]. Several studies have attested that cholesterol enhances the aggregation propensity of amyloidogenic peptides, including Aβ [99,100,101], α-syn [102,103], hIAPP [104], htt [105] and transthyretin [106]. Cholesterol-containing membranes have been shown to significantly accelerate amyloid-β aggregation and enhance its membrane-binding affinity [99,100,101]. Specifically, under time-lapse atomic force microscopy (AFM), the presence of cholesterol in 1-palmitoyl-2-oleoyl-*sn*-glycero-3-phospho-choline/1-palmitoyl-2-oleoyl-*sn*-glycero-3-phospho-L-serine (POPC/POPS) lipid bilayers led to the faster appearance of Aβ_42_ aggregates compared to non-cholesterol bilayers, implying an enhanced aggregation propensity [99]. Similarly, in 1,2-dimyristoyl-*sn*-glycero-3-phosphocholine (DMPC) vesicles, cholesterol increased the nucleation rate of Aβ_42_ peptides by catalysing the primary nucleation step [101]. Computational modelling also demonstrated that lipid membranes harbouring cholesterol manifested a stronger affinity to Aβ_42_, both in zwitterionic POPC [100] and anionic POPC/POPS [99] bilayers. Yu and Zheng [100] attributed the enhanced membrane-binding of Aβ peptides to cholesterol-induced alterations in the structure and dynamics of the lipid bilayer. Further, it was reported that cholesterol, together with the ganglioside content, promoted the conformational transition from an α-helix- to a β-sheet-rich structure [107].

In contrast, other substantial evidence supports a *protective* role for cholesterol against amyloid-β toxicity, describing cholesterol-rich bilayers to be less prone to destabilisation and perforation. Fernández-Pérez et al. [108] examined the impact of the cholesterol content on neuronal membranes, reporting that increasing membrane cholesterol levels increased the number of membrane-associated Aβ clusters but prevented membrane perforation and neurotoxicity. Additionally, high cholesterol content in neuroblastoma cell membranes prevented Aβ peptide binding, with the cholesterol content being inversely correlated with the membrane-perturbing effects of the Aβ oligomers [109]. Molecular dynamics (MD) simulations also support this notion, with cholesterol-rich raft model membranes being found to be more resistant to Aβ_42_ tetramer insertion than pure PC membranes [110]. Enhanced membrane resilience to amyloid-β is attributed to cholesterol-induced physicochemical changes in the bilayer, specifically a more packed and rigid environment that likely impedes membrane permeabilisation [108,109].

Similar to amyloid-β, the interplay between cholesterol, hIAPP and membranes is also complex [111]. Cholesterol has been reported to slow hIAPP aggregation and reduce dye leakage from PC and PS lipid vesicles, although this protective effect was overcome by high concentrations of anionic lipids [34]. MD simulations of POPC/POPS membranes also indicate that the addition of cholesterol caused a weaker insertion of hIAPP into the bilayer and made the membrane less vulnerable to hIAPP-induced poration [112]. However, whilst cholesterol suppressed membrane damage in raft-free anionic POPC/POPS vesicles, in raft-containing vesicles, cholesterol conversely exacerbated hIAPP fibrillisation and membrane leakage [104]. These findings suggest that the influence of cholesterol on hIAPP-mediated membrane leakage is to a significant extent attributed to its influence on the physicochemical properties of the lipid membrane, rather than via direct interactions with the peptide itself. By investigating different sterols, Zhang et al. [113] established that sterols which led to a more tightly packed membrane environment resulted in lower vesicle binding of hIAPP, decreased membrane leakage, and slower peptide assembly. This hypothesis is supported by other investigations showing that increased membrane rigidity caused by cholesterol impedes pore formation by melittin [114].

α-syn has a high affinity for cholesterol, mediated by residues 67–78 in the aggregation-prone non-amyloid-β component (NAC) domain of the protein [115]. In fact, cholesterol increased the propensity of α-syn to induce bilayer defects and was required for the formation of cytotoxic amyloid transmembrane pores [116]. However, cholesterol may also influence α-syn–bilayer interactions via alteration of the membrane environment, with contrasting effects. For instance, increasing the amount of cholesterol in DPPC vesicles weakened the binding of α-syn with the vesicles as a result of cholesterol blocking membrane defects [117]. As indicated by recent MD simulations, such an effect can be ascribed to a decrease in bilayer fluidity induced by cholesterol [102]. In agreement, other investigations have also reported that cholesterol inhibits the binding of α-syn to membranes comprising either zwitterionic or anionic lipids [103,118]. The impact of cholesterol on α-syn binding and fibrillation thus varies in different lipid environments.

Overall, while the relationship between the cholesterol membrane content and amyloid–membrane interactions remains an area of active investigation, the protective effects of cholesterol are often linked to its membrane-stabilising properties, which reduce the propensity for amyloidogenic peptides to penetrate the bilayer [119].

### 3.3. Polyphenols

Polyphenols are a class of phytochemicals with considerable structural diversity, essentially characterised by the presence of multiple aromatic rings with two or more phenol units. They are primarily found in herbal teas, fruits, and vegetables and are categorised according to the number of phenol rings and the elements connecting them [120]. Polyphenolic compounds show considerable promise as effective anti-amyloid agents and possess various biologically relevant properties, including antioxidant, anti-inflammatory, and antimicrobial effects. Several classes of polyphenols, including flavonoids, curcuminoids, and phenolic acids, have attracted intensive interest for their therapeutic potential for amyloid-related diseases due to their ability to inhibit the protein aggregation cascade [121,122,123,124,125,126]. Polyphenols that effectively antagonise amyloid formation share structural similarities. Such a capability is thought to be attributed to their phenolic rings, which disturb π-stacking interactions between monomeric units in aggregate species and form π–π interactions with aromatic residues present in amyloid-forming proteins [127,128]. However, the anti-aggregative effects of polyphenols on amyloid assembly are usually probed in vitro in aqueous solutions, which does not resemble the physiological environment in which such aggregates exist. This is especially significant in light of the substantial influence lipid membranes have on the aggregation cascade [129].

Polyphenols are able to interact with all the macromolecules, including lipids. They are generally localised close to the polar headgroups of the bilayer, just beneath the lipid–water interface. However, these interactions are greatly influenced by the structures of both the polyphenol and the lipid environment [130]. Numerous reports propose that the biological effects of polyphenols are attributable to their impact on membrane characteristics, such as fluidity [131,132].

#### 3.3.1. Curcumin

Curcumin is a widely researched lipophilic polyphenol abundantly found in the turmeric plant *Curcuma longa* [133]. Curcumin has attracted substantial interest for its potential therapeutic applications, including in proteinopathies [134]. Thus, curcumin potently interferes with the aggregation of the major amyloidogenic proteins, including amyloid-β [135,136], tau [137], α-syn [138], and hIAPP [139]. Notably, curcumin is reported to redirect the assembly of Aβ and α-syn towards the generation of non-toxic oligomers [136,140] and even disaggregate the preformed fibrillar aggregates of these proteins [135,137,140], thereby reducing the levels of neurotoxic species.

The breadth of curcumin’s interactions has led to speculation that in addition to its antioxidant, anti-inflammatory and anti-aggregative properties, the beneficial effects of this small molecule may also be membrane-mediated [141]. Due to its lipophilic nature, curcumin can distribute into cellular membranes, where it is reported to promote membrane cohesion and stability [142]. The orientation and distribution of curcumin within phospholipid bilayers is concentration-dependent. At lower concentrations, curcumin orients itself flat on the membrane surface, binding to the lipid–water interface through H- bonding with the phosphate headgroup, and acts as a protective steric barrier or “carpet”, reducing membrane fluidity and water permeability. Upon increasing the curcumin concentration, the molecule penetrates into the acyl chain region, where it fluidises the membrane and counters peptide insertion [141,143,144]. Curcumin thus reduced the rate and extent of Aβ insertion into anionic lipid monolayers, leading to decreased Aβ-induced bilayer disruption in a dose-dependent manner [145]. Furthermore, curcumin embedded into anionic bilayers reduced the accumulation of fibrillar Aβ oligomers on the membrane by almost 70% [146]. Along the same lines, research by Khondker and co-workers demonstrated that curcumin, by altering the structural parameters of the membrane, significantly reduced the formation of Aβ_25–35_ peptide plaques by an unsaturated anionic membrane model [147]. These and other investigations have provided further support for curcumin as a small-molecule polyphenol that inhibits amyloid self-assembly and membrane toxicity by a combination of membrane and protein interactions.

#### 3.3.2. Epigallocatechin Gallate (EGCG)

The green tea flavonoid EGCG is another well-studied polyphenol against amyloid protein self-assembly. Several studies have reported that EGCG effectively inhibits the aggregation of a number of disease-related peptides, including Aβ [148,149], tau [150], α-syn [122,151], hIAPP [152], and huntingtin [36], amongst others. Since this compound has exhibited promising therapeutic potential for multiple PMDs, EGCG has entered clinical trials for the treatment of neurodegenerative diseases. However, despite the positive outcomes observed in in vitro and animal models, few to no positive results have been reported to date in clinical trials [153].

EGCG is a gallic acid ester featuring seven hydroxyl groups distributed among three aromatic rings. Integration into lipid bilayers occurs mostly through its gallic acid ester group [148,154]. The presence of galloyl moieties in polyphenols enhances their lipophilicity, enabling galloylated catechins like EGCG to penetrate lipid bilayers and disturb the organisation of the tail region, in turn imparting morphological and fluidic alterations to membranes [131,155].

EGCG has been studied for its potential to inhibit amyloid aggregation and mitigate toxicity at lipid interfaces. Preincubation of Aβ_42_ with EGCG lessened the bilayer disruption of negatively charged vesicles, suggesting that EGCG attenuated the peptides’ membrane interaction [156]. This is consistent with recent MD simulations, which reported that in the presence of anionic membranes composed of POPC and POPG (1-palmitoyl-2-oleoyl-*sn*-glycero-3-phosphoglycerol) lipids, EGCG altered the binding dynamics between protofibrillar Aβ_42_ and the bilayer. Further, EGCG slowed the adsorption of Aβ_42_ and hindered its assembly, albeit less effectively than in a membrane-free environment. The numbers of contacts formed between EGCG and lipid bilayers were even greater than those between EGCG and Aβ_42_ protofibrils, implying that EGCG interacts preferentially and strongly with membranes [148]. In an analogous manner, EGCG associated primarily with the bilayer to weaken the H-bonding and hydrophobic interactions between protofibrillar α-syn and the membrane, hence limiting α-syn membrane-binding and enhancing membrane integrity [151]. Interestingly, EGCG retained its inhibitory effect on α-syn aggregation, as well as its capacity to reduce the concentration of oligomeric α-syn, in the presence of both zwitterionic and anionic liposomes [157]. Similarly, EGCG also prevented aggregation of the htt protein into amyloid fibrils even in the presence of vesicles made from the synthetic lipid POPC or from total brain lipid extract [158].

However, the efficacy of EGCG in membrane environments has not always been consistent. EGCG less effectively inhibited Aβ_40_ fibrillisation upon incubation with large unilamellar vesicles (LUVs), with PCPG anionic vesicles reducing the inhibitory efficacy of EGCG more markedly in comparison to zwitterionic PC vesicles [157]. Mass spectrometry experiments point to a likely partitioning of EGCG into the lipid bilayer, hence reducing the availability of this inhibitor to bind Aβ. In concordance, another study reported a significant reduction in the ability of EGCG to inhibit hIAPP fibrillogenesis in the presence of negatively charged dimyristoyl-phosphatidylglycerol (DPPG) lipid bilayers [159]. This was attributed to diminished π–π stacking interactions between EGCG and the hydrophobic binding sites of the peptide, which are sequestered within the lipid phase and thus inaccessible to EGCG. Taken together, these mechanistic insights highlight the possibility of reduced inhibitor efficacy in a lipid environment due to competing interactions between the inhibitor compound, the peptide and the membrane—hence further underscoring the influence of lipid membranes during therapeutic design.

### 3.4. Ubiquinones

Ubiquinones are a group of lipid-soluble antioxidant molecules located primarily in the membranes of Gram-negative bacteria and in the inner mitochondrial membranes of eukaryotic cells. Ubiquinones are composed of a quinone headgroup connected to a lipophilic isoprenoid chain of species-specific length, confining these molecules to lipid-rich environments [160]. In humans, the chain comprises ten isoprenoid units and is thus referred to as ubiquinone-10, or coenzyme Q10 (CoQ10). CoQ10 can be considered a mobile component of the mitochondrial electron transport chain, shuttling electrons in a ubiquinone (oxidised) and ubiquinol (reduced) redox cycle [161]. It therefore plays a significant role in cellular metabolism, participating in various aspects of mitochondrial energy production. Further, CoQ10 protects mitochondrial membrane lipids and proteins from free radical-induced oxidative injury [162,163]. While the exact position of Q10 in the bilayers remains to be fully elucidated, it is generally accepted that the isoprenoid chain is embedded within the mid-plane of the hydrophobic core, while the quinone ring alternates between the polar headgroup region and the mid-plane [164,165].

The influence of CoQ10 on the mechanical properties and permeability of lipid membranes may be compared to that of cholesterol, with studies suggesting that the inclusion of CoQ10 in the phospholipid leaflet imparts a concentration-dependent increase in the phospholipid order and a pronounced membrane-rigidifying effect [166,167]. In agreement with this, the levels of endogenous ubiquinones in bacteria increased dramatically under prolonged osmotic stress, implying a potential stability-enhancing role of ubiquinones within membranes [168]. This “stiffening” effect on lipid bilayers has been corroborated by other investigations employing biomimetic membranes [166,169].

Studies have reported that CoQ10 confers cyto-protection against Aβ through multiple mechanisms, including the inhibition of oxidative stress and increased expression of proteins related to neuronal cell survival [170], as well as membrane-mediated mechanisms delaying Aβ incorporation into membranes [171,172]. The protective effect of CoQ10 does not appear to be mediated by a simple chemical interaction with the Aβ peptide, since the simultaneous addition of CoQ10 and Aβ did not confer protection against Aβ toxicity in endothelial cells. However, pre-treatment of cultured cells with CoQ10 prevented Aβ-induced damage. CoQ10 was reported to prevent the incorporation of Aβ into the plasmalemma and mitochondrial membranes, reducing its accumulation within the cells. Additionally, CoQ10 decreased the production of ROS and prevented cell death [171,172]. Notably, the protective role of CoQ10 may be partially attributed to its ability to shield the membrane. It is hypothesised that CoQ10 acts as a “cellular armour”, impeding the insertion of Aβ into bilayers via steric hinderance, in turn reducing the formation of Aβ channels in the membrane. Furthermore, considering the oxidative stress [173] and mitochondrial dysfunction [174] hypotheses of AD, the antioxidant, neuroprotective, and anti-inflammatory properties of CoQ10 appear promising for the amelioration of the oxidative stress and neuroinflammation associated with AD [175]. This is further supported by evidence suggesting that serum CoQ10 levels may be inversely associated with the risk of developing dementia [176], and other reports demonstrating the protective effects of CoQ10 against Aβ neurotoxicity [170]. These findings underscore the need for further investigation into the mechanisms of action of CoQ10, particularly its ability to stabilise membranes and prevent the damaging insertion of other amyloidogenic peptides, whilst highlighting the critical role of bilayer stabilisation in mitigating amyloid toxicity.

## 4. Conclusions

In conclusion, the reviewed literature provides strong support for the notion that regulating the physical state of lipid membranes may be a promising therapeutic strategy to suppress the toxicity of amyloidogenic assemblies in PMDs. Considering the central role of membranes in amyloid pathology, the identification of small molecules that have the potential to modulate peptide–membrane interactions to (i) mitigate membrane-catalysed misfolding and assembly, and (ii) preserve membrane integrity, is an important step in the development of effective treatments for these debilitating disorders (Table 2). However, evidence also suggests that compound interaction with lipid membranes does not necessarily confer protection, as evidenced by EGCG, a well-established anti-amyloidogenic compound. The conflicting data and gaps in our understanding underscore the necessity for a broader investigation into the fundamental mechanisms by which these compounds interact with both peptides and membranes. Elucidating these complex interactions in future studies will be crucial for informing the design of more targeted and potent small-molecule therapeutics, which could address the membrane-mediated aspects of amyloid toxicity.

## Figures and Tables

**Table 1:** Selected amyloidogenic proteins and peptides with their associated human diseases (reviewed in [1,3,6]).

Peptide/Protein	Associated Disease/s
Amyloid-β peptide (Aβ)	Alzheimer’s disease
Atrial natriuretic factor	Atrial amyloidosis
Calcitonin	Medullary carcinoma of the thyroid
Fragments of immunoglobulin light chains	Light-chain amyloidosis
Huntingtin protein (htt)	Huntington’s disease (HD)
Human islet amyloid polypeptide (hIAPP)/amylin	Type-2 diabetes mellitus
Lysozyme	Lysozyme amyloidosis
N-terminal fragments of prolactin	Pituitary prolactinoma
Prion protein (PrP)	Creutzfeldt–Jakob disease Spongiform encephalopathies
Microtubule-associated protein (tau)	Alzheimer’s disease Frontotemporal dementia
Transthyretin	Senile systemic amyloidosis
α-synuclein (α-syn)	Parkinson’s disease Multiple system atrophy

**Table 2:** List of compounds and associated peptide/membrane models.

Compound	Protein/Peptide	Membrane Model	References
Squalamine	α-syn	DMPS vesicles DOPE/DOPS/DOPC (5:3:2) vesicles	[80,81]
Trodusquemine	α-syn	DMPS vesicles DOPE/DOPS/DOPC (5:3:2) vesicles Neuronal membranes (SH-SY5Y cells)	[85,87]
	Aβ, HypF-N	DOPE/DOPS/DOPC (5:3:2) vesicles DOPC/SM (2:1) vesicles with 1% CHOL and 1% GM1 Neuronal membranes (SH-SY5Y cells)	[87,88,89]
Cholesterol	α-syn	POPC, POPC/POPG, and POPC/POPE vesicles DPPC, DOPC and DOPC/SM vesicles DOPE/DOPS/DOPC (5:3:2) vesicles DOPC, DOPC/DOPE/DOPG (4:3:1) vesicles	[116]
	Aβ	POPC and POPC/POPS bilayers DMPC vesicles Raft model membranes Plasma membranes (HEK-293 cells, SH-SY5Y cells, and hippocampal neurons)	[99,100,101,108,109,110]
	hIAPP	DOPC and DOPC/DOPS bilayers DOPC and POPC lipid vesicles with DOPG, POPS or SM POPC/POPS (7:3) vesicles DOPC/DPPC (1:2) vesicles	[112]
Curcumin	Aβ	DMPC, DOPC, DMPG and POPC/DMPS (97:3) vesicles DMPG and DMPG/DMPC monolayers Neuronal membranes (SH-SY5Y cells)	[141,143,144,145]
EGCG	α-syn	POPC/POPG bilayers POPC and POPC/POPG vesicles	[151,157]
	Aβ	POPC/POPG (7:3) bilayers POPC, POPC/POPG and DMPC/DMPG vesicles	[156,157]
	hIAPP	DPPG bilayers	[159]
	htt	POPC vesicles Total brain lipid extract	[158]
CoQ10	Aβ	Endothelial cell membranes	[171,172]

*Abbreviations*: *CHOL*, cholesterol; *DMPC*, 1,2-dimyristoyl-*sn*-glycero-3-phosphocholine; *DMPG*, 1,2-dimyristoyl-*sn*-glycero-3-phospho-(1′-rac-glycerol); *DMPS*, 1,2-dimyristoyl-*sn*-glycero-3-phosphoserine; *DOPC*, 1,2-dioleoyl-*sn*-glycero-3-phosphocholine; *DOPE*, 1,2-dioleoyl-*sn*-glycero-3-phosphoethanolamine; *DOPG*, 1,2-dioleoyl-*sn*-glycero-3-phospho-(1′-rac-glycerol); *DOPS*, 1,2-dioleoyl-*sn*-glycero-3-phosphoserine; *DPPC*, 1,2-dipalmitoyl-*sn*-glycero-3-phosphocholine; *GM1*, monosialotetrahexosylganglioside; *POPC*, 1-palmitoyl-2-oleoyl-*sn*-glycero-3-phosphocholine; *POPE*, 1-palmitoyl-2-oleoyl-*sn*-glycero-3-phosphoethanolamine; *POPG*, 1-palmitoyl-2-oleoyl-*sn*-glycero-3-phospho-(1′-rac-glycerol); *POPS*, 1-palmitoyl-2-oleoyl-*sn*-glycero-3-phosphoserine; *SM*, sphingomyelin.
